# Does cup position differ between trabecular metal and titanium cups? A radiographic propensity score matched study of 300 hips

**DOI:** 10.1080/17453674.2020.1788290

**Published:** 2020-07-03

**Authors:** Inari Laaksonen, Natalie Hjelmberg, Kirill Gromov, Antti E Eskelinen, Ola Rolfson, Henrik Malchau, Anders Troelsen, Keijo T Mäkelä, Maziar Mohaddes

**Affiliations:** a Department of Orthopedics and Traumatology, Turku University Hospital, Turku, Finland, Finnish Arthroplasty Register, Helsinki, Finland;; b Swedish Hip Arthroplasty Register, Department of Orthopaedics, Institute of Surgical Sciences, Sahlgrenska University Hospital, Gothenburg, Sweden; c Department of Orthopaedic Surgery, Copenhagen University Hospital Hvidovre, Copenhagen, Denmark; d Danish Hip Arthroplasty Register, Aarhus, Denmark; e Coxa Hospital for Joint Replacement, Tampere, Finland, Finnish Arthroplasty Register, Helsinki, Finland;; f Harris Orthopedic Laboratory, Massachusetts General Hospital, Boston, USA

## Abstract

Background and purpose — The use of trabecular metal cups in primary total hip arthroplasty (THA) is increasing, despite the survival of Continuum cups being slightly inferior compared with other uncemented cups in registries. This difference is mainly explained by a higher rate of dislocation revisions. Cup malpositioning is a risk factor for dislocation and, being made of a highly porous material, Continuum cups might be more difficult to position. We evaluated whether Continuum cups had worse cup positioning compared with other uncemented cups.

Patients and methods — Based on power calculation, 150 Continuum cups from 1 center were propensity score matched with 150 other uncemented cups from 4 centers. All patients had an uncemented stem, femoral head size of 32 mm or 36 mm, and BMI between 19 and 35. All operations were done for primary osteoarthrosis through a posterior approach. Patients were matched using age, sex, and BMI. Cup positioning was measured from anteroposterior pelvic radiograph using the Martell Hip Analysis Suite software.

Results — There was no clinically relevant difference in mean inclination angle between the study group and the control group (43° [95% CI 41–44] and 43° [CI 42–45], respectively). The study group had a larger mean anteversion angle compared with the control group, 19° (CI 18–20) and 17° (CI 15–18) respectively.

Interpretation — Continuum cups had a greater anteversion compared with the other uncemented cups. However, the median anteversion was acceptable in both groups and this difference does not explain the larger dislocation rate in the Continuum cups observed in earlier studies.

Trabecular metal (TM) has become an increasingly popular implant material in both primary and revision total hip arthroplasty (THA) (Laaksonen et al. 2017, 2018). Its highly porous surface provides good initial stability and improves bone ingrowth (Bobyn et al. 1999, Beckmann et al. 2014). Continuum cups (Zimmer Biomet, Warsaw, IN, USA) with TM surface have showed higher revision rates than other uncemented cups after primary THA in some register studies mainly due to a higher dislocation rate (Laaksonen et al. 2018, Hemmilä et al. 2019).

Dislocation is one of the most common postoperative complications leading to revision surgery (AOANJRR 2017, Finnish Arthroplasty Register [FAR] 2017). Risk for recurrent dislocation and periprosthetic joint infection increases after revision surgery and therefore prevention of the first dislocation is vital (Ezquerra et al. 2017). Potential risk factors for dislocation are posterior approach, small femoral head size, fracture as the indication for surgery, female sex, and suboptimal acetabular cup positioning (Hailer et al. 2012, Zijlstra et al. 2017). Optimal cup positioning to avoid dislocation is traditionally defined by Lewinnek safe zones. According to this definition optimal cup inclination angle is 40° ± 10° and optimal anteversion angle is 15° ± 10° (Lewinnek et al. 1978. Slight modifications to optimize the stability have also been presented (Danoff et al. 2016). In particular, lower anteversion has been associated with increased dislocation rate (Seagrave et al. 2017a). We theorized that the higher dislocation rate for Continuum cups compared with other uncemented cups may be caused by suboptimal cup positioning due to difficulties in optimizing the acetabular cup position with this highly porous material.

In this observational multicenter cohort study, we analyzed whether there is a difference in acetabular implant positioning while using Continuum acetabular cups compared with other uncemented acetabular cups in primary total hip arthroplasty.

## Patients and methods

### Power calculation

Based on a previous publication describing cup positioning and its influence on dislocation, a power calculation (Biedermann et al. 2005) showed that a minimum cohort of 101 patients was needed in each group to detect a difference of 6 degrees abduction (n = 101) and 5 degrees in anteversion (n = 137), with 95% power and an α-error probability of 0.01. Recruiting 137 patients from each center will enable detection of 4 degrees difference in anteversion or inclination with a power of 95% (α = 0.05). To ensure study power in case of difficulties in the radiographic measurements 150 patients were included in both groups.

### Patients

**Figure 1.**
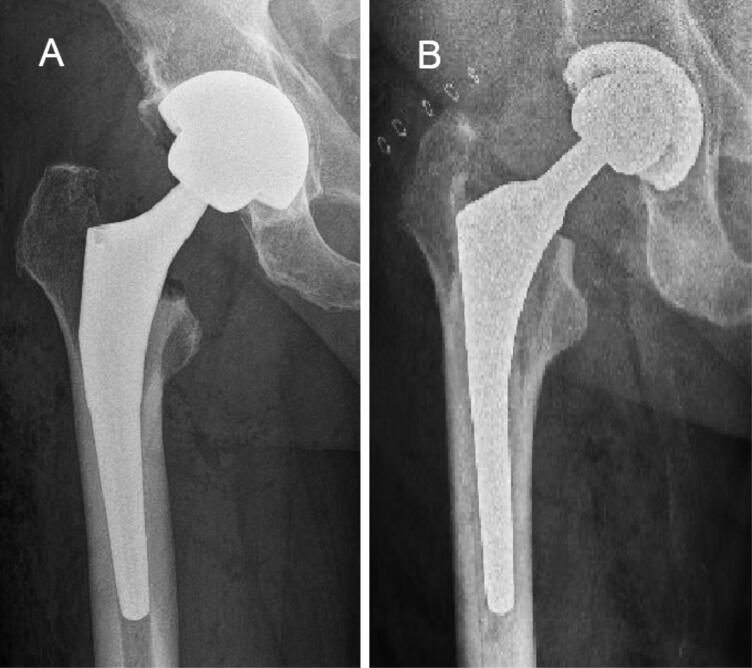
A Continuum cup (A) and a control group cup (B).

150 randomly selected primary total hip arthroplasty cases from the 3 joint replacement centers (Turku University Hospital, Turku, Finland; Coxa Hospital for Joint Replacement, Tampere, Finland; Varberg Hospital, Varberg, Sweden), using a porous Continuum tantalum cup were propensity score matched with 150 cases using a porous-coated titanium cup (the control group) from 1 center (Hvidovre, Copenhagen, Denmark) (Figure 1). The Continuum cup is used in most primary THAs in the study centers.

### Inclusion criteria

All patients were operated on for primary osteoarthrosis (OA) with a posterior approach between 2014 and 2017. Femoral head size used was 32 mm or 36 mm and all patients received an uncemented stem. All patients had a BMI between 19 and 35. Patients were matched using age, sex, and BMI (Table 1). All patients from the study centers fulfilling the inclusion criteria were collected for the matching process. No bilateral cases were included.

**Table 1.** Demographic and surgical data of study population. Values are number (%) unless otherwise specified

	Study group	Control group
Factor	n = 139	n = 150
Turku ^a^	50 (36)	
Coxa ^b^	42 (30)	
Varberg ^c^	47 (34)	
Hvidovre ^d^		150 (100)
Females	53 (38)	60 (40)
Age, median (SD)	65 (8.9)	65 (12)
BMI, median (SD)	28 (3.4)	27 (3.5)
Femoral head size		
32 mm	53 (38)	29 (19)
36 mm	86 (62)	121 (81)
Liner		
Hi-wall		150 (
Neutral	98 (70)	
Oblique	30 (22)	
10° elevation	11 (8)	

**^a^**Turku University Hospital, Turku, Finland

**^b^**Coxa Hospital for Joint Replacement, Tampere, Finland

**^c^**Varberg Hospital, Varberg, Sweden

**^d^**Hvidovre University Hospital, Copenhagen, Denmark

### Radiographic analysis

The Martell Hip Analysis Suite software (version 8.0.1.4.3, UCTech, University of Chicago, IL, USA) was used for radiographic measurements (Martell and Berdia 1997, Elson et al. 2015). The first postoperative anteroposterior radiograph of the pelvis (0–3 months) was used for measuring cup inclination and anteversion angles. All measurements in the study group was measured by 1 examiner (NH) during 2019. In the study group there was difficulties measuring abduction and anteversion angles in 11 hips due to suboptimal radiographs and all were excluded from further analysis.

### Statistics

Data selection and matching were applied using the R software (version 3.6.1; R Foundation for Statistical Computing, Vienna, Austria). A random sampling process (without replacement) was used to select 150 patients with Continuum cups. Propensity score matching, controlling for age, sex, and BMI, was used to select the control group of other uncemented cups. The propensity scores were estimated implementing a 1:1 nearest neighbor matching using logistic regression. The normal distribution of data was checked by generating histogram and qq plots and whenever the normal distribution did fulfil assumptions, a nonparametric Mann–Whitney test was used to compare groups (study vs. control). The sex and the operated side distributions in the 2 groups were checked by chi-square test. Data were presented as median (SD). A p-value < 0.05 was considered as statistically significant. Means and 95% confidence interval levels (CI) are presented.

### Ethics, registration, funding, and potential conflicts of interests

Ethical approval was obtained from the Local Ethical Review Board in Turku University Hospital (T01/003/18, date of issue May 8, 2018). This research received funding from the Southwestern Finland State Research Funding and from Turku University Hospital for radiograph transfer costs. All authors declare no conflict of interest.

## Results

There was no clinically relevant difference in mean inclination angle between the study group and the control group (43° [95% CI 41–44] and 43° [42–45], respectively) (Table 2). The study group had a larger mean anteversion angle compared with the control group, 19° (18–20) and 17° (15–18) respectively.

**Table 2.** Median cup angles and percentages of cups in the Lewinnek safe zone in the Continuum cup study group and the other uncemented cups control group

Factor	Study group	Control group
Cup angles, mean (95% CI)		
Inclination	43 (41–45)	43 (41–44)
Anteversion	19 (18–20)	17 (15–18)
Turku **^a^**		
Inclination	42 (39–46)	
Anteversion	15 (13–17)	
Coxa **^b^**		
Inclination	45 (42–49)	
Anteversion	24 (22–26)	
Varberg **^c^**		
Inclination	43 (40–46)	
Anteversion	19 (16–21)	
Hvidovre **^d^**		
Inclination		43 (41–44)
Anteversion		17 (15–18)
Cups in the safe zone, % (n)		
Inclination	67 (93)	81 (122)
Anteversion	76 (105)	67 (100)
Both	52 (72)	55 (82)

**^a–d^**See Table 1.

Center-wise inclination and anteversion angles were 43° (41–44) and 17° (15–18), respectively, for the control group and 45° (42–49) and 24° (22–26) for center 1, 43° (40–46) and 19° (16–21) for center 2, and 42° (39–45) and 15° (12–17) for center 3 in the study group.

**Figure 2.**
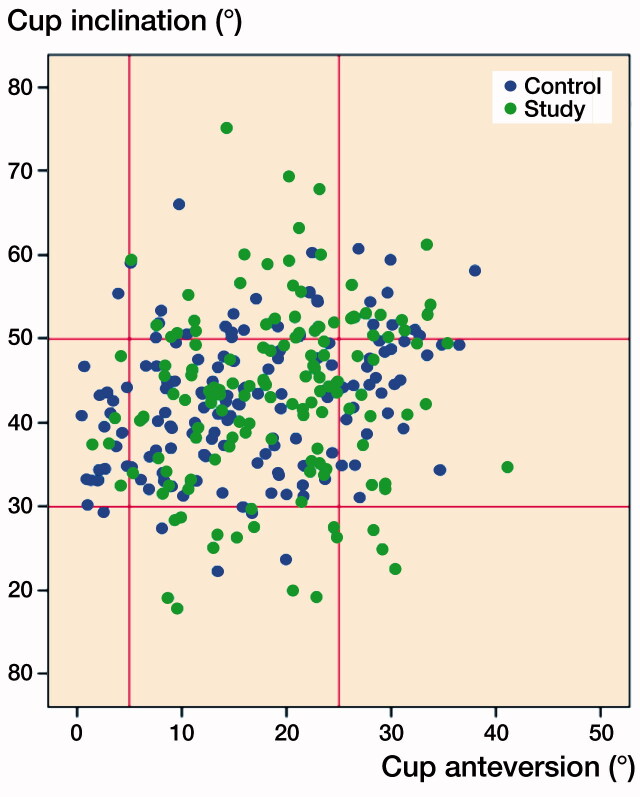
The scatter plot depicts Martell radiographic analysis, which compares the distribution of 2 groups in the safe zone.

Only 52% (n = 72) of the cups in the Continuum study group and 55% (n = 82) of the cups in the control group were in the Lewinnek safe zone when both inclination and anteversion angles were addressed (Figure 2, Table 2).

## Discussion

Recent register studies have presented higher revision rates for trabecular metal acetabular cups compared with other uncemented cups. This difference is mainly explained by higher dislocation rates. Acetabular component malpositioning is a known risk factor for dislocation in total hip arthroplasty. Trabecular metal is a highly porous material, which might make cups with a TM surface more difficult to implant in the desired position and therefore predispose to malpositioning. In this study we aimed to assess cup positioning in Continuum and other uncemented devices to evaluate potential malpositioning in Continuum cups.

Trabecular metal cups are a good option in demanding cases with large bone defects because of their good osteointegration qualities (Bobyn et al. 1999). The use of this highly porous material has increased significantly during the last decade in both primary and revision THA encouraged by the good results in short- to mid-term clinical studies (Jafari et al. 2010, Baad-Hansen et al. 2011, Mohaddes et al. 2015, Wegrzyn et al. 2015). Despite the improved osteointegration and stability, TM cups have had slightly higher revision rates in register reports, mainly due to revisions for dislocation (Hemmilä et al. 2019). A potential risk for bias is difference in patient selection, as TM surfaced cups are traditionally used in more demanding cases. However, the difference in revision rate is not likely to be fully explained by patient selection as TM surfaced primary cups are the primary acetabular components in primary THA in many centers (Laaksonen et al. 2018). The Continuum cup is the most common device used in primary THA in the study centers and therefore in this study there should not be bias in patient selection.

The higher revision rate for Continuum cups during short-term follow-up appears to be explained by higher dislocation revision rates (Hemmilä et al. 2019). One of the known risk factors for THA dislocation is acetabular component malpositioning (Biedermann et al. 2005). Too small anteversion and potential retroversion has traditionally been associated with higher dislocation risk (Seagrave et al. 2017a). There have been several attempts to generate optimal safe zones in cup positioning to minimize the dislocation risk, varying around 30°–45° for inclination and 5°–25° for anteversion (Callanan et al. 2011, Seagrave et al. 2017b). In our material there were no clinically relevant differences in the inclination angle between the study groups. Continuum cups had a higher anteversion angle than the control group; however, anteversion was acceptable in both groups and higher anteversion protects from dislocation than rather predisposes to it (Seagrave et al. 2017a). Even though our study group had a slightly higher median anteversion angle, in our material the median anteversion fitted within all suggested safe zones in both the Continuum study group and the control group. Nevertheless, there is no consensus on the optimal acetabular cup angles (Cotong et al. 2017). It is possible that due to earlier reports of the higher dislocation rate in Continuum cups surgeons are aiming for slightly greater anteversion in these cups than in other uncemented devices. This could explain the higher median anteversion observed in the study group. On the other hand, the use of elevated rim liners might also lead to a decrease in aimed anteversion as high anteversion combined with posterior elevation might lead to impingement.

As the anteversion was at an acceptable level and slightly larger in the study group than in the control group and the inclination comparable between the groups, difficulties in cup positioning are not likely to explain the higher dislocation rate for Continuum cups in earlier studies. 1 possible explanation is smaller coverage in neutral Continuum liners and smaller jumping distance, which predisposes to dislocation (Sariali et al. 2009). Oblique and elevated liners appear to assess this problem and reduce dislocation risk (Hemmilä et al. 2019). However, the long-term data on stabilizing liners’ effect on implant survival is limited and it is possible that stabilizing liners might cause posterior impingement and therefore predispose to anterior dislocation. Further, mean anteversion was higher in 1 of the study centers compared with the other 2 study centers. One potential explanation for this is that the ContinuumTM system had been used for only a short time in that center and at the beginning of the study period surgeons in this center were aiming for slightly greater anteversion as recommended in the system they used previously. However, the anteversion was within Lewinnek’s safe zone in all 3 study centers and greater anteversion in patients operated on with a posterolateral incision should protect from rather than predispose to dislocation.

Thus far TM components have demonstrated better survival compared with other uncemented cups in primary THA in only 1 register study. This study included other uncemented cups only from the same manufacturer and not the best performing uncemented acetabular devices (Matharu et al. 2018a). More long-term register data, especially including elevated and oblique liners, are needed to assess whether the overall revision risk for Continuum cups is lower compared with other uncemented devices at a later stage when aseptic loosening is the main reasons for revision. There has also been speculation based on a small clinical study that TM as a material might have some qualities protecting against PJI (Tokarski et al. 2015). Unfortunately, these results have not been reproducible in later studies and Matharu et al. (2019) advise clinicians to be cautious regarding such claims.

We acknowledge that our study has limitations. 1st, we were unable to reliably collect data with dislocation as the endpoint as some of the patients might have changed their treating hospital during the study time. Therefore, we could not study whether malpositioning predisposed to dislocation or try to create our own cup positioning safe zones according to dislocations. 2nd, due to the observational nature of this study, we were unable to randomly assign patients to the study or control group. However, to avoid potential bias, we have matched the groups by age, sex, and BMI, and all included patients were operated on for primary OA with a posterior approach, had an uncemented stem with femoral head size 32 mm or 36 mm, and BMI between 19 and 35. 3rd, we did not have data on surgeons’ experience that might affect to cup positioning and possibly cause bias. Further, another limitation is that the Martell system uses only pelvic AP radiographs when measuring the cup position. Hence there was a need to double check the lateral radiographs manually to ensure that acetabular components were not in retroversion.

In conclusion, Continuum acetabular components had greater anteversion compared with the other uncemented cups. However, anteversion was at acceptable level in both groups and this difference does not explain the larger dislocation rate in Continuum cups observed in earlier studies as greater anteversion protects from dislocation rather than predisposes to it.

The authors would like to acknowledge Emma Naucler and Ali Rafati for their help with the statistical analysis.
